# Attenuation of Myocardial Injury by HMGB1 Blockade during Ischemia/Reperfusion Is Toll-Like Receptor 2-Dependent

**DOI:** 10.1155/2013/174168

**Published:** 2013-11-24

**Authors:** Jan Mersmann, Franziska Iskandar, Kathrina Latsch, Katharina Habeck, Vera Sprunck, René Zimmermann, Ralf R. Schumann, Kai Zacharowski, Alexander Koch

**Affiliations:** ^1^Department of Anaesthesia, Intensive Care Medicine and Pain Therapy, University Hospital Frankfurt, Goethe University, Theodor-Stern-Kai 7, 60590 Frankfurt am Main, Germany; ^2^Department of Trauma Surgery, University Hospital Gießen and Marburg GmbH, 35043 Marburg, Germany; ^3^Institute for Microbiology and Hygiene, Charité Medical Center, 12203 Berlin, Germany

## Abstract

Genetic or pharmacological ablation of toll-like receptor 2 (TLR2) protects against myocardial ischemia/reperfusion injury (MI/R). However, the endogenous ligand responsible for TLR2 activation has not yet been detected. The objective of this study was to identify HMGB1 as an activator of TLR2 signalling during MI/R. C57BL/6 wild-type (WT) or TLR2^−/−^-mice were injected with vehicle, HMGB1, or HMGB1 BoxA one hour before myocardial ischemia (30 min) and reperfusion (24 hrs). Infarct size, cardiac troponin T, leukocyte infiltration, HMGB1 release, TLR4-, TLR9-, and RAGE-expression were quantified. HMGB1 plasma levels were measured in patients undergoing coronary artery bypass graft (CABG) surgery. HMGB1 antagonist BoxA reduced cardiomyocyte necrosis during MI/R in WT mice, accompanied by reduced leukocyte infiltration. Injection of HMGB1 did, however, not increase infarct size in WT animals. In TLR2^−/−^-hearts, neither BoxA nor HMGB1 affected infarct size. No differences in RAGE and TLR9 expression could be detected, while TLR2^−/−^-mice display increased TLR4 and HMGB1 expression. Plasma levels of HMGB1 were increased MI/R in TLR2^−/−^-mice after CABG surgery in patients carrying a TLR2 polymorphism (Arg753Gln). We here provide evidence that absence of TLR2 signalling abrogates infarct-sparing effects of HMGB1 blockade.

## 1. Introduction

Toll-like receptors being the most prominent class of innate immunity receptors are the first line of defence against invading pathogens. Beyond host defence, these receptors also have a role in the majority of sterile inflammatory conditions. We and others have shown that antibody blockade [1], pharmacological preconditioning [2, 3], or genetic ablation [4, 5] of toll-like receptor 2 (TLR2) signalling is protective in a mouse model of myocardial ischemia and reperfusion (MI/R). The underlying mechanisms reported so far include augmented phosphoinositide-3-kinase/Akt signalling, increased preservation of Connexin 43 gap junctions, reduced proapoptotic signalling, reduced proinflammatory cytokine, and chemokine release with subsequently reduced leukocyte infiltration [1–5]. It is, however, unclear which of its proposed endogenous ligand(s) causes TLR2 activation in the setting of MI/R. 

High mobility group box 1 (HMGB1) as a nuclear protein has two DNA-binding domains and is involved in stabilization of nucleosomes and regulation of gene expression [6]. As a cytosolic protein it is involved in the promotion of autophagy [7], and as an extracellular protein it is either passively released from necrotic cells [8] or actively secreted by immune cells upon proinflammatory stimulation [9]. Since HMGB1 release elicits or augments inflammatory reactions [10, 11] it was often regarded as the prototype damage associated molecular pattern (DAMP, as opposed to PAMP, pathogen associated molecular patterns, which are typical TLR ligands). This view has however changed, when in a number of studies highly purified HMGB1 failed to exert proinflammatory cytokine-like functions (reviewed in [12]). It was therefore concluded that HMGB1 might act as a chaperone potentiating or modulating the effects of proinflammatory mediators. 

A role for HMGB1 in MI/R was established in a study by Andrassy et al. that, in line with the proinflammatory, cytokine concept for HMGB1 showed increased myocardial damage when HMGB1 was administered and decreased necrosis when HMGB1 was competitively blocked by its nonfunctional fragment Box A [13]. Both effects could not be observed in the absence of the receptor for advanced glycation end products (RAGE), which was the first identified receptor for HMGB1 in neuritis and macrophages [14, 15]. However, besides RAGE, other receptors including TLR2, TLR4, and TLR9 have been identified as HMGB1 receptors [16–18], and the utilization of these receptors might be largely different between cell types and tissues [19]. 

In the present study, we therefore tested the hypothesis that administration or pharmacological blockade of HMGB1 affects myocardial injury after MI/R and that these effects are dependent on TLR2. Besides its putative adverse effects in MI/R, it must be noted, that during recovery from myocardial infarction exogenous HMGB1 injection has been shown in a number of studies to prevent adverse remodelling and improve cardiac function [20–22]. The present preclinical study was, however, conducted to identify HMGB1 as a ligand/cofactor responsible for TLR2 activation during the acute phase of MI/R. 

There is a high frequency of heterozygous carriers of a nonfunctional TLR2 polymorphism (Arg753Gln) in the Caucasian population [23]. Carriers of the Arg753Gln mutation are at a higher risk for coronary restenosis after percutaneous transluminal coronary angioplasty (PTCA) [24]. To identify a possible relation between HMGB1 release and presence of the Arg753Gln polymorphism in MI/R we quantified serum HMGB1 concentrations in patients undergoing elective coronary artery bypass graft (CABG) surgery. Patients had been recruited for a trial investigating toll-like receptor polymorphisms in cardiac surgery. A subgroup analysis was performed in carriers of the Arg753Gln polymorphism undergoing elective CABG surgery, which were compared to age- and sex-matched controls from the study collective.

## 2. Materials and Methods

### 2.1. Animals

Male C57BL/6JRj Wildtype (WT) and TLR2^−/−^ mice (B6.129-TLR2^tm1kir^/J, The Jackson Laboratories, Bar Harbor, Maine, USA), back-crossed to the C57BL/6J-background for 9 generations, were used. The age of animals used in this study ranged between 5 and 7 months. The animals were housed under specified pathogen-free conditions and had access to standard laboratory animal chow and water *ad libitum*. All procedures were performed in accordance with the *Guide for the Care and Use of Laboratory Animals* published by the United States National Institutes of Health and were approved by the local government authorities (approval reference number F91/53, Regierungspräsidium Darmstadt, Germany).

### 2.2. Myocardial Ischemia/Reperfusion (MIR)

 Experimental myocardial ischemia was performed as described previously [25, 26]. Mice were anesthetized with an intraperitoneal (i.p.) injection of pentobarbital (Narcoren 90 mg/kg, Merial GmbH, Hallbergmoos, Germany). For analgesia animals received buprenorphine s.c. (0.05 mg/kg) before and 8 hrs after the intervention. Mice were orally intubated and ventilated using a MiniVent (Hugo Sachs Elektronik-Harvard Apparatus, March-Hugstetten, Germany) with a tidal volume of 9 *μ*L/g bodyweight and a frequency of 110/min. F_i_O_2_ was set to 0.5. The front aspect of the heart was visualized by a left thoracotomy in the 4th intercostal space. The left anterior descending (LAD) coronary artery was ligated using 7-0 suture (Seralene, Serag-Wiessner, Naila, Germany), the ends of which were passed through a short PE tube, pulled tight, and held into place by a microserrefine. Ischemia was confirmed by the myocardium turning notably pale. After 30 minutes of ischemia, tension of the thread was released and reperfusion was confirmed visually. After wound closure animals were weaned from the respirator. The tracheal tube was removed once spontaneous breathing was sufficient. Oxygen was administered via a face mask until animals spontaneously removed themselves from the ventilator. After 24 hours of reperfusion, animals were anesthetized as described above. Blood was taken from the inferior caval vein with a heparinised syringe, the ligation was retightened, and EvansBlue dye (Sigma-Aldrich, Munich, Germany) was injected via the right ventricle. 

Animals were injected i.p. one hour prior to ischemia with either Vehicle (Veh, 5% DMSO in phosphate buffered saline), HMGB1 Box A (300 *μ*g, LPS-free, HMGBiotech, Milano, Italy) or recombinant HMGB1 (600 *μ*g, LPS-free, eBioscience, Frankfurt, Germany).

### 2.3. Infarct Size Determination

Evans Blue-stained hearts were excised and immediately cut into five equal slices. Pictures of these slices were taken before and after incubation with p-nitro blue tetrazolium (0.5%, 25 min, 37°C, Sigma-Aldrich, Munich, Germany). Infarct size (IS) and area at risk (AR) were planimetrically quantified using SigmaScan Pro image measurement software (SPSS Inc., Chicago, IL, USA).

### 2.4. Cardiac Troponin T

Blood samples were centrifuged for 7 minutes at 7000 ×g. Plasma was stored at −80°C until assayed. Samples were diluted 1 : 10 in whole blood of untreated WT animals and cardiac troponin T was quantified using the Cardiac Reader (Roche, Mannheim, Germany), according to the manufacturer's instructions.

### 2.5. Leukocyte Count

Leukocyte infiltration was quantified in paraformaldehyde-embedded 5 *μ*m sections stained with hematoxylin and eosin (H&E). For each animal 10 microscopic fields with a magnification of 20x, covering 3.2 mm^2^ of the area at risk were analyzed.

### 2.6. Real-Time rt-PCR

Real-Time PCR was performed using SYBR Green incorporation on a StepOnePlus Real-Time PCR system (Applied Biosystems, Weiterstadt, Germany) according to the manufacturer's instructions. Samples were analyzed in duplicate. Results are presented as x-fold expression of the ANAR of WT animals using the ΔΔCt method. Primers used were HMGB1 (forward 5′-TGC GTC TGG CTC CCG CTC TC-3′, reverse 5′-AGT TGA TTT TCC TCC GCG AGG CAC-3′), RAGE (forward 5′- CAC TTG TGC TAA GCT GTA AGG G-3′; reverse 5′-CAT CGA CAA TTC CAG TGG CTG-3′), TLR4 (forward 5′-GCC TTT CAG GGA ATT AAG CTC C-3′; reverse 5′-GAT CAA CCG ATG GAC GTG TAA A-3′), TLR9 (forward 5′-ACA ACT CTG ACT TCG TCC ACC-3′; reverse 5′-TCT GGG CTC AAT GGT CAT GTG-3′), 18S (forward 5′-CAC GGG AAA CCT CAC CCG GC-3′, reverse 5′-CGG GTG GCT GAA CCG CCA CTT-3′).

### 2.7. Immunohistochemistry

RAGE expression was assessed by standard immunohistochemistry protocols using goat antimouse primary antibody (# AF1179, R&D systems, Wiesbaden, Germany) and rabbit antigoat secondary antibody (# HAF017, R&D systems, Wiesbaden, Germany). Antibody binding was visualized by diaminobenzidine (DAB). DAB-positive area was expressed as fraction of the total area of 10 microscopic fields at a magnification of 20x covering 3.2 mm^2^.

### 2.8. Plasma HMGB1

Samples were analyzed by ELISA in microtiter plates. Wells were coated with 50 *μ*L/well of anti-HMGB1/HMG1 (Upstate Biotechnology, Lake Placid, NY) at 1.5 *μ*g/mL. Following overnight incubation at 4°C, plates were blocked with 1% BSA in PBS. Standard and serum samples were added and incubated for two hours at room temperature. After washing, 50 *μ*L biotinylated anti-HMGB1 (R&D Systems, Abingdon, UK), 0.75 *μ*g/mL, was added and incubated for one hour at room temperature. After washing, streptavidin peroxidase was added and incubated for one hour at room temperature. After washing, 3,3′,5,5′-tetramethylbenzidine dihydrochloride (TMB) was added. Reactions were stopped after 15 minutes with H_2_SO_4_ (1 M) and read spectrophotometrically at 450 nm.

### 2.9. Patient Recruitment

The clinical trial was conducted to investigate the role of TLR polymorphisms in cardiac surgery. Inclusion and exclusion criteria were published elsewhere [27]. Briefly, patients undergoing elective cardiac surgery (coronary artery bypass graft (CABG) and/or valve surgery (VS) including replacement and reconstruction) were included. Inclusion criteria are as follows: patients had to be of age 18 or older, and had elective cardiac surgery, on cardiopulmonary bypass (CPB). Exclusion criteria are as follows: cardiac surgery performed without CPB, history of diseases affecting the HPA axis, or systemic or local treatment with glucocorticoids within 30 days before surgery. The study was approved by the local ethical review committee (University Hospital Dusseldorf) and carried out in compliance with the principles established in the Helsinki Declaration and all further amendments. Written consent was obtained. DNA was extracted from whole blood by commercial kits (QIAmp, Qiagen, Hilden, Germany). Genotyping for TLR2 SNP Arg753Gln (rs5743708) was performed by melting curve analysis employing FRET probes and the Lightcycler (Roche Diagnostics, Mannheim, Germany) as described previously [28]. From the study collective 5 patients heterozygous for the TLR2 polymorphism, Arg753Gln were compared with 10 age- and sex-matched controls. Plasma HMGB1 levels were determined before and on day 3 after surgery. Patient demographics are presented in Table 1.

### 2.10. Statistics

Statistical analysis was performed using Prism software (GraphPad Software Inc., La Jolla, CA). Results are presented as mean ± SEM of n observations. All datasets underwent normality testing. Comparisons between groups were made using Mann-Whitney *U* or Student *t*-tests, where appropriate. *P* < 0.05 was considered significant.

## 3. Results

### 3.1. HMGB1 Antagonist Box A Reduced Myocardial Damage

**Figure 1:**
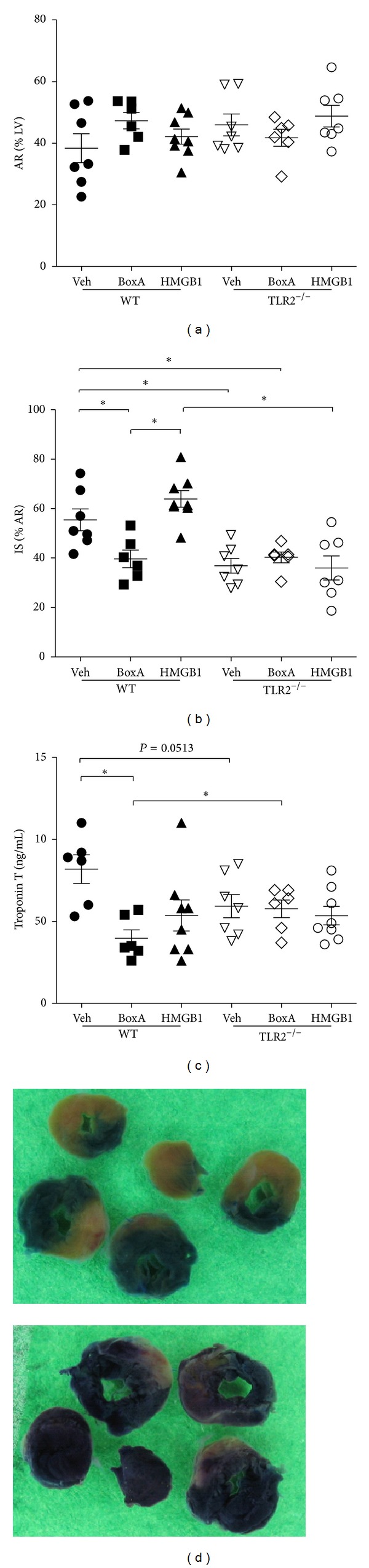
Quantification of myocardial damage after ischemia (30 min) and reperfusion (24 hrs). (a) The area at risk relative to the left ventricle (AR%LV) was not different between experimental groups. HMGB1 Box A reduced (b) infarct size relative to the AR (IS%AR) and (c) Troponin T plasma levels compared to vehicle-treated (Veh) WT animals. TLR2^−/−^ mice showed reduced myocardial damage compared to WT mice, whereas Box A had no protective effect in these animals. Injection with HMGB1 did not aggravate myocardial necrosis in both genotypes. (d) Hearts perfused with EvansBlue (EB, above) were cut into five equal sections, photographed, and incubated with p-nitro-blue-tetrazolium (NBT, below) for the quantification of AR and IS. *P < 0.05.

The area at risk (AR) relative to the area of the left ventricle (LV) was similar in all experimental groups (Figure 1(a)). Treatment with Box A, a nonfunctional fraction and competitive antagonist of HMGB1, reduced infarct size (IS), and troponin T (TnT) plasma levels as compared to vehicle-treated WT mice (Figures 1(b) and 1(c)). In TLR2^−/−^ mice, which as expected showed reduced myocardial damage after MI/R, Box A treatment had no effect on infarct size or TnT levels. 

### 3.2. Injection with HMGB1 Did Not Aggravate Myocardial Necrosis

Contrariwise, animals were treated with purified HMGB1 injection to aggravate myocardial injury. However, HMGB1 injection had no effect on infarct size or TnT levels after MI/R in both WT and TLR2^−/−^ (Figures 1(a) and 1(c)).

### 3.3. Reduced Leukocyte Infiltration after Box A Treatment

**Figure 2:**
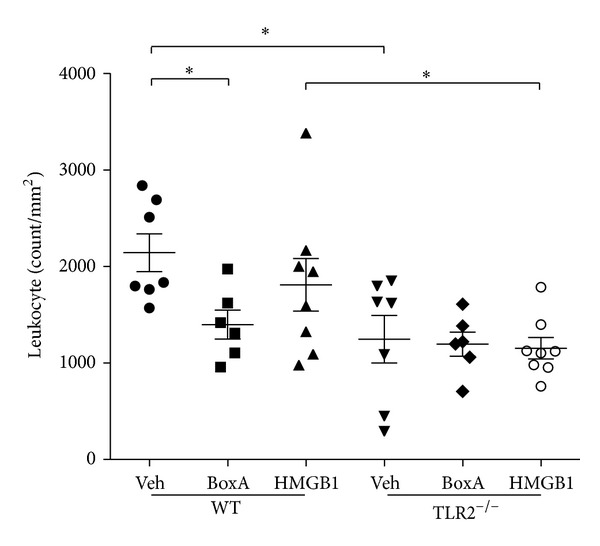
Leukocyte infiltration after myocardial ischemia (30 min) and reperfusion (24 hrs). Box A reduced leukocyte infiltration in WT animals, while it had no effect in TLR2^−/−^ animals. HMGB1 injection did not affect leukocyte infiltration in both WT and TLR2^−/−^. *P < 0.05.

Injection with Box A reduced leukocyte infiltration into the AR in WT animals compared to Veh-treated controls (Figure 2). Concomitant to its failure to aggravate myocardial injury, injection with purified HMGB1 did also not augment leukocyte infiltration in WT animals. Leukocyte infiltration in TLR2^−/−^ was not affected by either Box A or HMGB1 treatment. 

### 3.4. Expression Levels of HMGB1 and Its Receptors RAGE, TLR4, and TLR9 after MI/R

**Figure 3:**
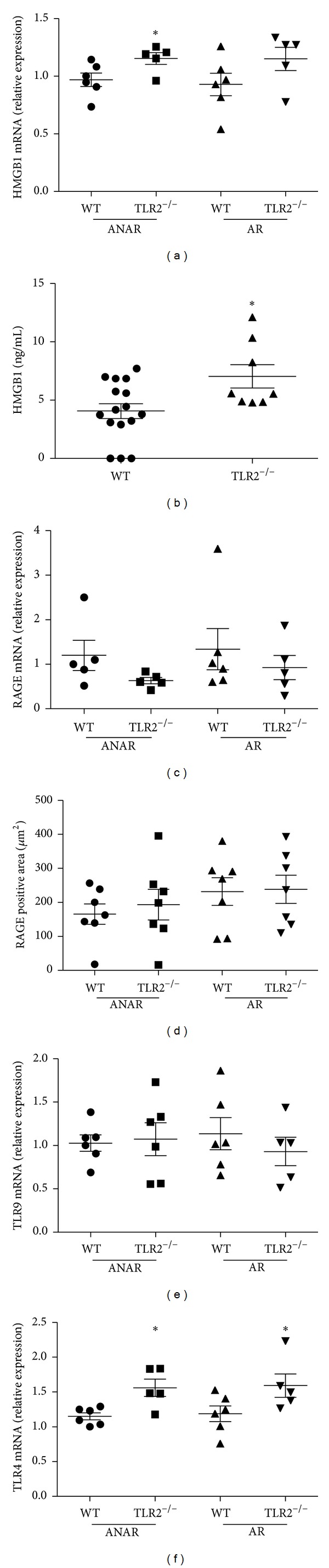
Expression levels of HMGB1 and its receptors after MI/R (30 min/24 hrs). (a) A moderate up-regulation of HMGB1 mRNA can be detected in the area not at risk (ANAR) of TLR2^−/−^ animals, (b) accompanied by increased HMGB1 plasma levels. Expression levels of RAGE (c, d) or TLR9 (e) were not significantly different between genotypes while TLR2^−/−^ animals showed an up-regulation of TLR4 mRNA (f) compared to WT in ANAR and AR. *P < 0.05, ANAR area not at risk, AR area at risk.

HMGB1 mRNA was moderately increased in the ANAR of TLR2^−/−^ animals after MI/R as compared to WT (Figure 3(a)). This was accompanied by significantly higher HMGB1 plasma levels compared to WT (Figure 3(b)). The expression of RAGE, the main receptor for HMGB1 in several cell types, was however not different between genotypes (Figures 3(c) and 3(d)), neither was the mRNA expression of TLR9 (Figure 3(e)). TLR4, another receptor for HMGB1, showed increased mRNA expression in TLR2^−/−^ animals as compared to WT (Figure 3(f)).

### 3.5. Patients Heterozygous for the Arg753Gln TLR2 Polymorphism Displayed Increased HMGB1 Release after CABG Surgery

No significant differences could be detected between the carriers of the Arg753Gln polymorphism and age- and sex-matched controls in the following variables (Table 1): body mass index (BMI), duration of surgery, cardiopulmonary bypass time, aortic cross-clamp time, or release of the myocardial isoform of creatine-kinase (CK-MB) in relation to total CK release (CK-MB [%CK_total_]).

**Figure 4:**
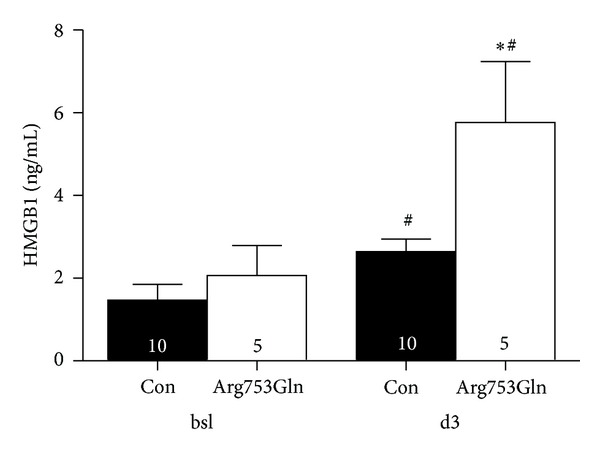
Patients undergoing coronary artery bypass surgery with extracorporeal circulation displayed increased HMGB1 serum levels on day 3 after surgery compared to baseline (^#^ *P* < 0.05 versus bsl). Heterozygous carriers of the less-functional Arg753Gln TLR2 polymorphism showed significantly higher levels compared to healthy controls (con). *n* = 5–10, *P < 0.05 versus con.

All patients undergoing CABG surgery with extracorporeal circulation showed increased HMGB1 plasma levels on day 3 after surgery as compared to baseline (Figure 4). Induction of HMGB1 release was however significantly more pronounced in carriers of the Arg753Gln polymorphism. 

## 4. Discussion

The results from the present study identify a relation between HMGB1 and TLR2-signalling in myocardial ischemia and reperfusion. 

Consistent with former observations [13], competitive antagonism of HMGB1 via its subunit Box A ameliorated myocardial damage after MI/R in WT animals, which was accompanied by decreased leukocyte infiltration. Neutrophil infiltration is one of the hallmarks of reperfusion injury [29], perpetuating tissue injury in the acute phase of reperfusion while being a prerequisite for myocardial wound healing and scar formation. In neutrophils, however, RAGE has only a minor role in the promotion of HMGB1-induced NF-*κ*B activation as compared to TLR2 and TLR4 [16]. Furthermore, preconditioning with HMGB1 renders THP-1 monocytes less responsive to stimulation with TLR2 agonist lipoteichoic acid, a mechanism independent of RAGE [30]. 

In TLR2^−/−^ animals, which as previously reported [4], showed reduced myocardial necrosis and leukocyte infiltration, no further protection could be observed after treatment with Box A, which would have been expected if HMGB1 signalling was solely RAGE-dependent in MI/R [13]. This is even more highlighted by the observation, that endogenous HMGB1 release is significantly increased in these animals after MI/R. A compensating alteration of RAGE or TLR9 expression in TLR2^−/−^ myocardium could be excluded, while a moderate up-regulation of TLR4 became evident in these animals.

The recombinant HMGB1 preparation used in this study could not augment myocardial necrosis in WT animals, which is, however, in line with recent evidence questioning the perception that HMGB1 itself serves as a proinflammatory mediator [31–34]. Unlike the recombinant HMGB1 used in the pertinent study by Andrassy et al. showing aggravation of myocardial necrosis after treatment with HMGB1 [13], the recombinant HMGB1 preparation used in the present study was a commercially available lipopolysaccharide-free preparation. This preparation was also tested in HL-1 cardiomyocytes wherein it failed to induce proinflammatory mediators (Tumour necrosis factor *α*, Interleukin-6, Chemokine (C-X-C motif) ligand 2, data not shown). Accordingly, no effects of HMGB1 treatment were observed in WT or TLR2^−/−^ animals. 

The question whether HMGB1 functions as a cytokine itself or as a cofactor/chaperone for other DAMPs cannot be answered by the present study. The data provided here, however, allow for the conclusion that successful infarct-sparing therapies blocking HMGB1 in MI/R might be dependent on the presence of TLR2. The latter might be of particular interest for the development of therapeutic strategies targeting HMGB1, because less functional TLR2 polymorphisms are frequently found in the Caucasian population (9.4%) [23] and in patients undergoing cardiac surgery (3.8%) [27]. TLR2^−/−^ animals show elevated HMGB1 plasma levels 24 hours after MI/R as compared to WT. Similarly, heterozygous carriers of the Arg753Gln polymorphism displayed significantly increased serum HMGB1 levels on day 3 after CABG surgery as compared to control patients, suggesting that this group of patients might respond differently to HMGB1-targeting therapy. This is even more intriguing since elevated HMGB1 plasma levels have been identified to be independently associated with mortality and residual ventricular function in ST-segment elevation myocardial infarction treated by percutaneous coronary intervention (PCI) [35, 36], and carriers of the Arg753Gln polymorphism are at higher risk for coronary restenosis after MI and PCI [24]. 

## 5. Conclusions

In conclusion this study contributes to our understanding of the role of HMGB1 in myocardial ischemia and reperfusion. Besides the known involvement of its receptor RAGE, we here provide evidence for a role of TLR2 in mediating effects of endogenous HMGB1 released during acute myocardial ischemia and reperfusion.

## Figures and Tables

**Table 1:** Patient demographics.

	Control	Arg753Gln	*P*
*n*	10	5	
Male	9/10	5/5	NS
Age	69.5 (48–80)	66.0 (53–79)	NS
BMI	30.5 (24.4–33.9)	27.0 (23.5–32.9)	NS
Duration of surgery (min)	249.5 (180.0–320.0)	225.0 (170.0–270.0)	NS
Cardiopulmonary bypass time (min)	160.5 (94.0–233.0)	123.0 (110.0–163.0)	NS
Aortic cross-clamp time (min)	58.0 (30.0–82.0)	51.0 (42.0–58.0)	NS
CK-MB (%CK_total_)	7.3 (2.2–23.0)	13.9 (3.8–22.8)	NS

Total numbers, median (min–max), Fisher's test, Mann-Whitney test, BMI: body mass index, CK: creatine kinase, NS: not significant.
